# Prevention of Sexually Transmitted Infections and Associated Factors Among Night School Students in Bahir Dar City, Ethiopia

**DOI:** 10.1177/1178633720927374

**Published:** 2020-06-08

**Authors:** Yilkal Tiruneh Ayele, Mulusew Andualem Asemahagn, Tadesse Awoke

**Affiliations:** 1 Department of Maternal and Child Health, Amhara Regional Health Bureau, Bahir Dar, Ethiopia; 2 School of Public Health, College of Medicine and Health Sciences, Bahir Dar University, Bahir Dar, Ethiopia; 3 Institute of Public Health, College of Medicine and Health Sciences, University of Gondar, Gondar, Ethiopia

**Keywords:** STIs, knowledge, practice, factors, night school students, Bahir Dar, Ethiopia

## Abstract

**Background:**

Sexually transmitted infections (STIs) continued to be a global public health concern, primarily among adolescents in poor socioeconomic countries. This study was aimed to assess knowledge, practice, and associated factors of night school students to prevent STIs in Bahir Dar city, Northwest Ethiopia.

**Methods:**

An institution-based cross-sectional study was conducted among 422 students randomly selected from night schools. Data were collected using a pretested structured questionnaire. Data were entered, cleaned, and analyzed using SPSS version 16 software. Descriptive statistics were used to describe study variables. A multivariable logistic regression analysis was used to identify factors associated with knowledge about STI and its prevention. The strengths of associations were described using odds ratio at 95% confidence interval and a *P*-value of less than 0.05.

**Results:**

A total of 420 consented students were enrolled into the study. More than half, 420 (57.9%), and three-fourths, 332 (79%), of the students were women and married. Only 24.8% and 12.4% of the students had good knowledge and practice on the prevention of STIs, respectively. Age, educational status, information access, school health education, and consistent use of condoms were factors associated with the knowledge of students about STIs. Similarly, age, sex, knowledge about STIs, and discussion with families were factors of STI prevention practice among night school students.

**Conclusions:**

Giving special attention to awareness creation, information access, discussion with families, and improving family/individual income is vital to prevent STIs and its impact among night school students.

## Background

Sexually transmitted infections (STIs) are infections caused by multiple organisms and mainly transmitted through vaginal, oral, and anal sexual contacts.^1^ The STIs could also be transmitted from mother to child during pregnancy and through blood products or tissue transfer.^1,2^ Sexually transmitted infections remain a major global health problem, majorly among the young population.^3,4^ An estimated 340 million new cases of curable STIs occurred every year worldwide between 15 and 49 years of age.^1^ About 1 million people contract STIs every day, and 50% of them aged between 15 and 24 years.^5,6^

Despite several interventions, prevention of STIs remains one of the sustainable development goal agendas. Less attention to the prevention of STIs, unsafe sexual practices, and low community and students’ awareness levels contributed more to the presence of high STIs in developing countries.^7-9^

Based on the study findings from southern Ethiopia, only 36.0% of school students had good knowledge about the prevention of STIs. More than half (52.2%) of students had multiple sexual partners, where 11.0% had sexual intercourse with commercial sex workers.^10^ Similarly, in the study findings from Gondar and Bahir Dar city, 39.0% and 65.2% of school students had good knowledge about the prevention of STIs, respectively.^11,12^ Having multiple sexual partners, watching sexual films, abortion, conflicts between couples, dropping from their education, substance abuse, and conflict with families are commonly observed phenomena in the study area.

In Ethiopia, night school students are people who are unable to continue their regular education due to resource limitation, lack of supporters, and work conditions.^13^ Most of the night school students in the study area were house workers, daily laborers, drivers, merchants, and guardians. Hence, they are expected to have limited awareness about the prevention of STIs. These conditions might affect the prevention of STIs among night school students.^14-16^

Moreover, little is known about the knowledge and practice of night school students to prevent STIs in Ethiopia, particularly in the Amhara Region. Therefore, this study was aimed at assessing the knowledge and practice of night school students on the prevention of STIs in Bahir Dar city, Northwest Ethiopia. The findings of this study will be important to prevent acquiring STIs among night school students and improve school performance.

## Methods

### Study design and period

An institution-based cross-sectional study was conducted in May 2014 among 422 students selected from night schools.

### Study area and settings

Bahir Dar city is located about 565 km to the Northwest of Addis Ababa, a capital city of Ethiopia. It has 21 kebeles (9 urban and 12 rural) with a total population of 288 200 (male = 140 603, female = 147 597). There are 14 high schools and 56 elementary schools (public and private) with a total of 64 682 (58 264 days and 6418 night) students. Seventeen schools have night school programs that have a total of 6418-night school students.

### Sample size determination and sampling techniques

All the night school students whose age ⩾15 years were study population to this study. The sample size was determined using Epi Info version 3.5.4 with the following assumptions: the existing knowledge and practice of students (p) is 50% (no previous study among night school), 95% confidence interval (CI), 5% margin of error, and 10% contingency. Then, the final sample size became 422. From 17 night schools, 4 (Fasilo high school: 309 students, Dona Ber elementary school: 828 students, Kulkual Meda elementary school: 307 students, and Meskerem 16 elementary school with 416 students) were randomly selected by lottery method. The sample size for each school was allocated proportionally. A sampling frame was developed from the roster of students in each school and a simple random sampling was used to select each study participant.

### Data collection tools and procedures

A structured self-administered questionnaire was used to collect data by assuming they can understand the questions as they are above 15 years, the questions are in their local language, and STIs are sensitive issues to respond by a face-to-face interview. The questionnaire was developed by referring to different papers on prevention of STIs.^8-11,17-20^ It was first prepared in English, translated to Amharic (local language) and back to English to check its consistency. It was pretested among night school students outside the actual study area to check its clarity, consistency, and ease of understanding each question. Four diploma nurses and 1 supervisor (instructor from Bahir Dar Health Science College) collected data at night times when they were in school to make the process easy, keep their privacies, and reduce influences from the families and couples. Data collectors distributed the questionnaires after informing students and getting consent to participate.

The knowledge of students about the prevention of STIs was assessed using 25 knowledge questions. Similarly, the prevention practice of the students was assessed using 20 practice-related questions. The values of each question were recorded and computed to give a single cutoff value, which is a percent value. Thus, the cutoff value is not simply based on a response from a single question. Students who answered above 50% of the 25 knowledge and 20 practice questions correctly were considered as having good knowledge and practice about the prevention of STIs, respectively. There are no standard cutoff values used to measure knowledge and practices due to variations in the situation of study subjects and the importance of questions asked to get the intended idea. Therefore, the reasons for using 50% cutoff values in our study are the absence of nationally or internationally agreed up cutoff value to measure knowledge and practices. In addition, the real condition of night school students (absence of information sources, time shortage to access information as most are daily laborers, poor attention to the prevention of STIs, lower-income level). Therefore, we assumed that a 50% cutoff value might tell us something about the knowledge and practice of night school students on the prevention of STIs in the study area.

### Data quality assurance and analysis

Questionnaire pretesting, training of data collectors and supervisors, supportive supervision of data collectors, and checking data completeness/consistency were data quality activities. Data were entered, edited, and analyzed using the SPSS version 16 statistical software. Various descriptive statistics such as frequencies, percentages, and proportions were computed to describe study variables. Bivariate and multivariable logistic regression analyses were used to identify factors associated with students’ knowledge and practice about the prevention of STIs. The strength of association between dependent and independent variables was described using odds ratio at 95% confidence interval (CI).

## Ethical Clearance

The Ethical Review Board of the College of Medicine and the Health Sciences, University of Gondar, reviewed the proposal document and gave an ethical clearance. Supporting letters were obtained from the Amhara Regional Health and State Education bureaus. Students and concerned stakeholders were informed about study objectives, data collection procedures, and data confidentiality issues prior to the actual data collection. Verbal informed consent was taken from each school director and student. For students below 18 years, informed consent was taken from their parents/caregivers. Participation was fully voluntary based including the right to withdraw from the study without any preconditions.

## Results

### Socio-demographic characteristics of students

A total of 420-night school students completed the questionnaires with a response rate of 99.5%. More than half, 243(57.9%), of the students were women and the mean age of the students was 21 ± 4.52 years. Students below the age category of 15 to 19 years constitute 51.7% of the studied students. There is only 1 elementary school in the sample with a population of 416 students; the high schools in the study had 1544 students constituting 21.2% of the population. The result shows that 36.9% of respondents were in grades 1 to 4. Of the students, 217 (51.7%), 65 (15.5%), and 138 (32.9%) were employed, merchants, and unemployed, respectively. Most of the night school students 381 (90.7%) were Orthodox Christians and 332 (79.0%) were unmarried. Less than half, 195(46.4%), of the students completed 5 to 8-grade levels, and 51.0% of them were from noneducated parents. About 171 (40.7%) students were from families earning <500 Ethiopian Birr/ETB/(20$) monthly (Table 1).

**Table 1.** Socio-demographic characteristics of night school students in Bahir Dar town, 2014 (n = 420).

Variables	Response	Frequency	Percent
Age in years	15-19	217	51.7
	20-24	136	32.4
	25+	67	16.0
Sex	Male	177	42.1
	Female	243	57.9
Occupation	Employed	217	51.7
	Merchant	65	15.5
	Unemployed	138	32.9
Religion	Orthodox	381	90.7
	Muslim	25	6.0
	Others	14	3.3
Marital status	Married	81	19.3
	Single	332	79.0
	Divorced	7	1.7
Grade level	1-4 grade	155	36.9
	5-8 grade	195	46.4
	9-10 grade	70	16.7
Parents’ education level	All are educated	88	21.0
	One of them educated	118	28.1
	All are not educated	214	51.0
Average monthly family income	Less than 500 ETB (20$)	171	40.7
	501-1000 ETB (20$-40$)	129	30.7
	1001-1500 ETB (40$-60$)	74	17.6
	Above 1500 ETB (>60$)	46	11.0
Average individual monthly income	No income	130	31.0
	Less than 300 ETB (12$)	64	15.2
	301-600 ETB (12$-24$)	49	11.7
	601-900 ETB (24$-36$)	45	10.7
	901 + ETB (>36$)	132	31.4

Abbreviation: ETB, Ethiopian Birr.

### Variables on students’ knowledge and practice to prevent STIs

The overall knowledge score of students about the prevention of STIs was 104 (24.8%). Most, 350(83.3%), of them heard information about STIs from different sources: 332 (79.0%) and 301 (71.7%) of them knew STI signs/symptoms and transmission routes, respectively. Most, 382 (93.3%), of the students reported that STIs are preventable. About 269 (64.0%) and 244 (58.0%) students reported that open discussion with partners and families is important to prevent STIs. On the contrary, the overall STI prevention practice of students was 12.4%. Only 33.6% and 43.8% of the students got health education on STIs from their schools and health workers before a year. Only (22.4% and 19.5%) students discussed with their partners and families on the prevention of STIs (Table 2).

**Table 2.** Night school students’ knowledge and practice to prevent STIs in Bahir Dar town, 2014 (n = 420).

Variables	Response	Frequency	Percent
Overall knowledge about STIs	Good	104	24.8
	Poor	316	75.2
Overall STI prevention practice	Good	52	12.4
	Poor	368	87.6
Heard about STIs	Yes	350	83.3
	No	70	16.7
Knew about transmission routes of STIs	Yes	301	71.7
	No	119	28.3
Knew the signs and symptoms of STIs	Yes	332	79.0
	No	88	21.0
Think STIs are preventable	Yes	382	93.3
	No	38	9.0
Knew risk factors of STIs	Yes	375	89.3
	No	45	10.7
Belief discussion with partner/s can prevent STIs	Yes	269	64.0
	No	151	36.0
Belief discussion with families can prevent STIs	Yes	244	58.0
	No	176	42.0
Got school health education on STIs	Yes	141	33.6
	No	279	66.4
Got information from health workers	Yes	184	43.8
	No	236	56.2
Discussed with a partner on STIs	Yes	94	22.4
	No	326	77.6
Discussed with family on STIs	Yes	82	19.5
	No	338	80.5
Had sex with the influence of alcohol/drugs	Yes	36	8.6
	No	384	91.4
Had sex without a condom	Yes	36	8.6
	No	384	91.4
Using condom in every sexual contact	Yes	124	29.5
	No	296	70.5

Abbreviation: STIs, sexually transmitted infections.

### Factors affecting students’ knowledge about the prevention of STIs

Education level, individual income, school health education, information on STIs from health workers, discussion on STIs with families, and consistent uses of condom in every sexual intercourse were statistically significant variables (*P* < .05) to students’ knowledge on prevention of STIs. Also, students aged between 20 and 24 years were more likely to have better knowledge about the prevention of STIs compared with students aged between 15 and 19 years. Similarly, the odds of having good knowledge on the prevention of STIs was higher among students above grade 5 compared with students with grades 1 to 4.

In addition, students who had their own income were more likely to be knowledgeable about the prevention of STIs compared with students who had no own income. Moreover, students who had open discussions with their families were more likely to be good at knowing STI-preventive mechanisms compared with the counterpart students (Table 3).

**Table 3.** Bivariate and multivariable logistic regression analysis on the knowledge of night school students to prevent STIs in Bahir Dar city, 2014 (n = 420).

Variables	Response	Knowledge		COR 95% CI	AOR 95% CI
		Good	Poor		
Age in years	15-19	40	177	1	1
	20-24	44	92	2.12 [1.29-3.48]	1.83 [1.05-3.18]
	25+	47	20	1.88 [1.01-3.52]	0.72 [0.58-1.89]
Sex	Male	53	124	1.61 [1.03-2.51]	1.32 [0.78-1.98]
	Female	51	192	1	1
Students’ education level	Grades 1-4	22	133	1	1
	Grades 5-8	57	138	2.50 [1.45-4.31]	2.81 [1.57-5.04]
	Grades 9-10	25	45	3.36 [1.73-6.53]	2.14 [1.03-4.45]
Has individual income	Yes	79	206	1.69 [1.02-2.61]	1.75 [1.01-3.10]
	No	25	110	1	1
Got school health education on STIs	Yes	58	83	3.54 [2.23-5.61]	2.14 [1.65-3.56]
	No	46	233	1	1
Got information from the health workers	Yes	70	114	3.65 [2.28-5.84]	3.74 [2.24-6.25]
	No	34	202	1	1
Have a boyfriend or girlfriend	Yes	51	125	1.47 [0.94-2.30]	0.56 [0.45-1.85]
	No	53	191	1	1
Had discussion with families on STIs	Yes	30	52	2.06 [1.19-3.56]	1.85 [1.14-2.46]
	No	74	264	1	1
Had sex without condom	Yes	12	24	0.63 [0.30-1.31]	0.48 [0.25-1.12]
	No	92	292	1	1
Had confidence in condom use	Yes	49	75	2.86 [1.86-4.55]	2.33 [1.40-3, 90]
	No	55	241	1	1

Abbreviations: AOR, adjusted odds ratio; CI, confidence interval; COR, crude odds ratio; STIs, sexually transmitted infections.

### Factors affecting students’ practice on the prevention of STIs

Age, sex, knowledge about STIs, and open discussion with partners and families were statistically significant factors (*P* < 0.05) to students’ practice on the prevention of STIs. Students aged between 20 and 24 years were more likely to have good practice in the prevention of STIs compared with students aged between 15 and 19 years. The odds of having good STI prevention practice was higher among male students compared with the counterpart students. Students who had open discussions with their partners and families were more likely to have good STI prevention practices than students who had no practice of open discussion with their families and partners (Table 4).

**Table 4.** Bivariate and multivariate logistic regression analysis on the practice of night school students to prevent STIs in Bahir Dar city, 2014 (n = 420).

Variables	Response	Practice		COR 95% CI	AOR 95% CI
		Good	poor		
Age in years	15-19	13	204	1	1
	20-24	22	114	3.03 [1.47-6.24]	2.34 [1.11-4.93]
	25+	17	50	5.34 [2.43-11.70]	2.92 [1.25-6.78]
Sex	Male	37	140	4.02 [2.13-7.59]	2.94 [1.50-5.80]
	Female	15	228	1	1
Occupation	Employed	19	198	0.57 [0.29-1.10]	0.25 [0.12-1.09]
	Merchant	13	52	1.48 [0.68-3.19]	0.86 [0.53-2.13]
	Unemployed	20	118	1	1
Has individual income	Yes	41	244	1.89 [0.94-3.81]	1.24 [0.68-2.11]
	No	11	124	1	1
Knew STI transmission routes	Yes	44	257	2.36 [1.03-5.21]	1.86 [1.23-3.05]
	No	8	111	1	1
Had an open discussion on STIs with partners	Yes	44	225	3.50 [1.60-7.64]	2.95 [1.32-6.60]
	No	8	143	1	1
Had a discussion with families on STIs	Yes	40	204	2.68 [1.36-5.27]	1.35 [1.24-3.56]
	No	12	164	1	1

Abbreviations: AOR, adjusted odds ratio; CI, confidence interval; COR, crude odds ratio; STIs, sexually transmitted infections.

## Discussion

Sexually transmitted infections continued to be a major public health problem worldwide, mainly in resource-limited countries. This study revealed that only 24.8% and 12.4% of night school students had good knowledge and practice of STIs prevention, respectively. This prevention knowledge is lower compared with study findings from Turkish Cypriot and Brazil, where most of the students (91.25% and 86.3%) had good knowledge of prevention of STIs, respectively.^21,22^ This might be due to differences in sociodemographic, infrastructure, personal behavior, information access, economic status, culture, school environment, and student-family interactions.

Similarly, this finding was found smaller compared with study findings from Southern Ethiopia (36%)^10^ and Northern Ethiopia (39%).^11^ The probable explanation for this variation might be poor attention from concerned bodies, inadequate information sources, poor family, and partner discussion in Bahir Dar city. This could have resulted from a higher number (51.7%) of 15 to 19 years population, and only 46.2% and 40.7% of students had individual and family income, respectively, in the study area (Table 1). In addition, the Ethiopian culture might play an important role in lowering the knowledge and practice of night school students to prevent STIs. Most of the time, the Ethiopian culture does not appreciate open discussion between adolescents and their parents/caregivers, particularly on STIs and reproductive health,^11,12,20^ because families usually believe that open discussion with adolescents will initiate them to practice sexual intercourse during their school age. This makes students shiny to express their opinion and will make everything hidden to their families, even will start silent sexual relationships with either school students or the outside community members. Parallel to this fact, most of the night school students in Ethiopia are from a disadvantaged background and unable to access adequate information and care about their health. Some (8.6%) (Table 2) were even forced to have unsafe sex with commercial sex workers, substance abusers, and rich people to get money and continue their education. This is supported by evidence from various areas,^1,6-10^ where the younger population groups acquired STIs due to poor knowledge and socioeconomic issues.

Students aged between 20 and 24 years were more likely to have good knowledge of prevention of STIs compared with students aged between 15 and 19 years (Table 3). This is in line with study findings from Ghana and German.^17,18^ The probable explanation for this might be students’ exposure to various information sources such as friends, media, the Internet, and books.

The students of grade 5 and above were relatively knowledgeable about the prevention of STIs than students of grades 1 to 4 (Table 3). This is similar to study findings from Ghana,^11,18,20^ where the students’ educational level was a significant factor to have knowledge about STIs. It is true that as the education level increased, the probability of exposure to various health information sources would also be increased.

Individual income played an important role in students’ knowledge of STI prevention. Students having their own income were more likely to have a better knowledge of STI prevention compared with their counterpart students (Table 3). It is similar to study findings from Malaysia,^17^ where higher economic class students were more knowledgeable about the prevention of STIs. If students have their own income, they may have access to various information sources, including advanced technologies to search for evidence compared with fewer income students.

Health education given by the health workers was found to be statistically significant to improve students’ knowledge of STI prevention (Table 3). This might be related to students’ attention or information preference to the information given by the health workers than the other sources. Students grouped in ⩾20 years age category had relatively better STI prevention practices compared with students in 15 to 19 years age category (Table 4). It is supported by study findings from Ethiopia,^10-12^ and abroad,^1,6,7^ which might be due to elders might have sexual intercourse and participate in STI prevention activities. Equally, they might have concerns about STIs and start a discussion with others as a result of age maturity and community acceptance to discuss STIs.

In this study, male students were more likely to participate in STI prevention than female students (Table 4). Although the Ethiopian culture does not support discussion on STIs and reproductive health between families and younger, men relatively discuss better with their peers compared with female students. Our culture also supports male dominance.^1,11,12,20^

## Conclusions

In this study, the knowledge and practice of night school students on STIs were poor compared with the previous findings. Sociodemographic, access and type of health information sources, and personal behaviors (discussion with families/partners, and consistent use of condom) were significant factors to knowledge on prevention of STIs, whereas, age, sex, knowledge about prevention of STIs, and open discussion with partners/families were factors associated with the practice of STI prevention. Improving information sources, discussions with families and income are key actions to prevent STIs and their impacts among night school students in Bahir Dar city.

## Limitations of the Study

The absence of adequate literature on knowledge and practice of night students to prevent STIs and not being supported by qualitative methods might affect the quality of findings and discussion. Using a self-administered questionnaire might also affect study findings due to social desirability bias and lower education levels to understand and respond to the questions.
